# Dynamic Pulse Buckling of Composite Stanchions in the Sub-Cargo Floor Area of a Civil Regional Aircraft

**DOI:** 10.3390/ma13163594

**Published:** 2020-08-14

**Authors:** Andrea Sellitto, Francesco Di Caprio, Michele Guida, Salvatore Saputo, Aniello Riccio

**Affiliations:** 1 Department of Engineering, University of Campania Luigi Vanvitelli, via Roma 29, 81031 Aversa, Italy; salvatore.saputo@unicampania.it (S.S.); aniello.riccio@unicampania.it (A.R.); 2 CIRA—Italian Aerospace Research Centre, via Maiorise snc, 81043 Capua, Italy; f.dicaprio@cira.it; 3 Department of Industrial Engineering, University of Naples Federico II, p.le Tecchio 80, 80125 Naples, Italy; michele.guida@unina.it

**Keywords:** dynamic pulse buckling, composite stanchion, FE analysis, nonlinear analysis, crashworthiness

## Abstract

This work is focused on the investigation of the structural behavior of a composite floor beam, located in the cargo zone of a civil aircraft, subjected to cyclical low-frequency compressive loads with different amplitudes. In the first stage, the numerical models able to correctly simulate the investigated phenomenon have been defined. Different analyses have been performed, aimed to an exhaustive evaluation of the structural behavior of the test article. In particular, implicit and explicit analyses have been considered to preliminary assess the capabilities of the numerical model. Then, explicit non-linear analyses under time-dependent loads have been considered, to predict the behavior of the composite structure under cyclic loading conditions. According to the present investigation, low-frequency cyclic loads with peak values lower than the static buckling load value are not capable of triggering significant instability.

## 1. Introduction

Buckling is an instability phenomenon typical of “thin” structures (characterized by at least one very small dimension compared to the others). Usually, buckling has been considered a purely static phenomenon. The classic example is that of the Euler beam in which a beam stuck at one end is loaded from the tip to the other, with a compressive load lower than the limit of elasticity of the material. Theoretically, if the force is perfectly centered and the beam is free of imperfections, the latter would remain in equilibrium under the action of any load. In order for the instability to occur, it is mandatory to destabilize the beam by means of an external action, immediately removed. After the perturbation, three cases can occur, depending on the applied load: The beam returns to the initial equilibrium configuration (stable equilibrium); the beam moves to a new equilibrium condition different from the initial one (indifferent equilibrium); the beam moves away indefinitely from the initial equilibrium configuration (unstable equilibrium). The so-called buckling load is the lowest of the loads for which equilibrium is indifferent. However, buckling can also be caused by loads that vary over time [1,2,3]. The application of a time-dependent axial load to a beam, which then induces lateral vibrations and can eventually lead to instability, is something that has been investigated by many authors [4,5,6,7,8,9,10].

Generally, dynamic buckling depends on prescribed dynamic loads, and is distinguished between buckling from oscillatory loads and buckling from transient loads, so two forms of dynamic buckling are classified: Vibration and pulse buckling.

Vibration buckling is characterized by oscillating loads producing amplification of the vibrations up to unacceptable values, and, in the absence of damping or nonlinear effects, a phenomenon similar to vibration resonance is predicted. In the vibration buckling, the force producing the instability is considered as a parameter multiplying the displacement, while in the vibration resonance, the load exciting the motion is evaluated as an applied force.

In pulse buckling, the presence of applied transient loads generates much larger and unacceptable amplitudes than the static buckling load of the bar as a result of the plastic response or large-deformation post-buckled state. The pulse buckling is characterized by buckling increasing rather than oscillating, and the modes produce wavelengths larger than the Euler wavelength for a given load.

The dynamic buckling of the column in an aircraft landing stanchion is a great deal and, in this work, the structural behavior of a composite floor beam subjected to low-frequency cyclic load conditions has been investigated. Indeed, the structural limits of beam-like structures is driven by their stability, rather than by their strength. The loss of stability induced by static loads is a well-known phenomenon, extensively investigated [11]. However, the loads that actually act on the structures are characterized by dynamic behavior, which can result in dynamic instability [12,13,14,15]. For these reasons, more detailed studies on the dynamic buckling phenomenon became mandatory in order to accurately predict the structural limits of the components subjected to realistic loads [16].

Dynamic buckling has a relatively recent history. One of the first research studies on dynamic buckling can be found in [17], where a theoretical solution considering a simply supported rectangular plate subjected to varying floor loads over time has been developed. In [18], a criterion that relates dynamic buckling to the duration of the load has been introduced. The effects of high-intensity and short-duration loads have been studied in [19]. According to the investigation, long-lasting critical dynamic buckling loads may be of less intensity than the corresponding static buckling loads.

In [20], a numerical and experimental investigation was performed on composite laminated shells by correlating their natural vibration frequencies and mode shapes with the buckling loads and modes. Moreover, parametric studies on the dynamic instability of carbon-fiber-reinforced plastic (CFRP) cylindrical shells were introduced in [21]. Different parameters were considered, such as the shape and the duration of pulse loading, and the initial geometric imperfection of the structure. According to the study, the dynamic load factor (DLF), defined as the ratio between the dynamic and the static buckling loads, is higher than that if short-pulse-duration loadings are used. Then, as the pulse duration increases, the DLF decreases, being lower than that in the vicinity of the natural frequency of the structure.

Other works have focused on the investigation of dynamic buckling on damaged composite structures. In [22], the nonlinear dynamic pulse buckling of a plate with embedded delamination was numerically investigated, and the DLF was computed. Different types of pulse loadings (sinusoidal, exponential, and rectangular) and different delamination sizes and depths were considered. Moreover, in [23], the dynamic instability of damaged composite components was studied, and the effects of the damage and its position were assessed. The damage was numerically simulated by reducing the stiffness of the components.

Different studies on the dynamic buckling induced by impact loading can be found in the literature [24,25,26]. In [27], the dynamic buckling of an elastic cylindrical shell subjected to axial impact was presented, and the influence of the boundary condition on the critical velocity of the impactor was assessed. In [28], the dynamic pulse buckling of composite laminated beams subjected to impact in the axial direction was investigated. In particular, the influence of different beams parameters (transverse and axial inertias, shear deformation, cross-section’s rotational inertia, and axial shortening) on the beams’ pulse buckling response was assessed.

Interesting insight on the dynamic instability can be found in [29]. A very detailed study was presented by the authors, which categorized the phenomenon according to the nature of the external load, whose direction can vary depending on the deformation of the structure, classifying the dynamic buckling problems as flutter or pulse buckling.

In this work, detailed numerical analyses have been presented, aimed to investigate the dynamic buckling response of the composite stanchion, in the finite element code Abaqus environment (release 2019, Dassault Systèmes Simulia Corp: Providence, Rhode Island, RI, SUA). Preliminary finite element models have been presented and compared, considering different in-plane and through-the-thickness element dimensions, different element types, and different material formulations, in order to find the model that more faithfully reproduces the investigated phenomenon. The latter has been used for the subsequent numerical investigation: The static stability limit of the composite stanchion (identified by its static critical buckling load) has been found; then, three different low-frequency cyclic load conditions have been considered (below, close, and above the static critical buckling load). In Section 2, the test cases are described, while in Section 3, the numerical analyses are introduced, including preliminary analyses aimed to determine the most suitable numerical model able to describe the dynamic phenomenon, and the results are critically discussed.

## 2. Test Case Description

**Figure 1:**
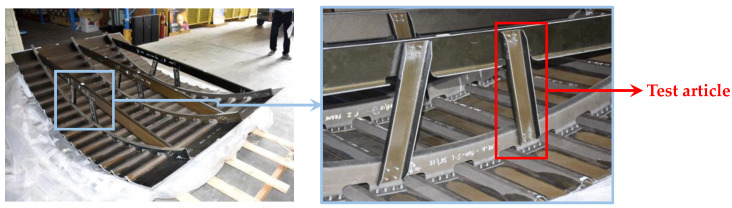
Location of the stanchion in a typical fuselage section.

The investigated test case, which has been previously validated in [30,31,32], is representative of the stanchion of the cargo floor of a regional aircraft of general aviation [33]. Images of the stanchions and their location in a typical fuselage section are reported in Figure 1.

**Figure 2:**
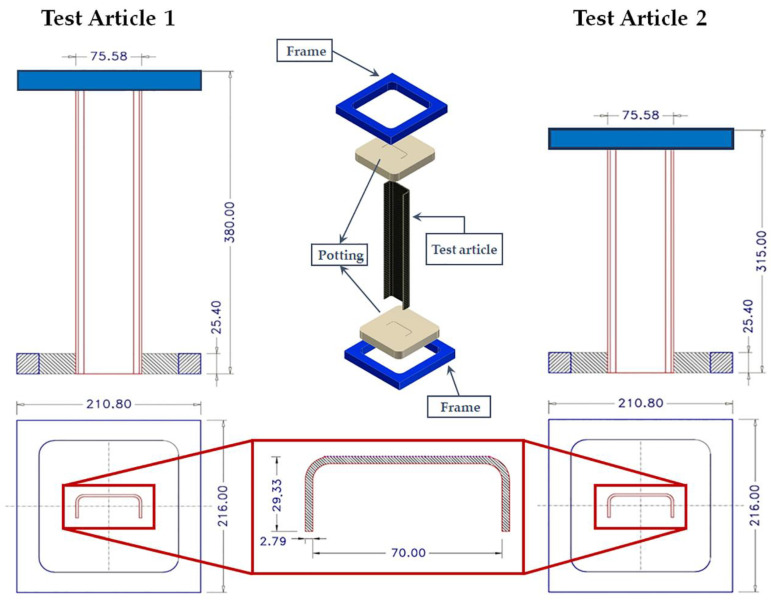
Geometrical description and boundary conditions.

Two different test articles, with different geometrical characteristics, have been considered. In particular, Test Article 1 is 380 mm long, while the length of Test Article 2 is 315 mm. More details on the test cases’ geometry and boundary conditions can be found in Figure 2. According to Figure 2, both sides of the specimens have been potted. A compressive displacement has been applied on one side, while the opposite side has been fixed.

The stanchion material is a composite made of carbon fibers and epoxy matrix at high polymerization temperature. The mechanical properties of the composite lamina are reported in Table 1, where ρ is the nominal density, th is the lamina thickness, E is Young’s modulus, G is the shear modulus, υ is Poisson’s ratio, X_t_ and X_c_ are respectively the longitudinal tensile and compressive strengths, Y_t_ and Y_c_ are respectively the transversal tensile and compressive strengths, and S_c_ is the shear strength. The stacking sequence of the laminate is [45; −45; 90; −45; 45; 0; 0; 0; 0; 0; 45; −45; 90; −45; 45].

## 3. Results and Discussion

### 3.1. Mesh Convergence Analysis

**Figure 3:**
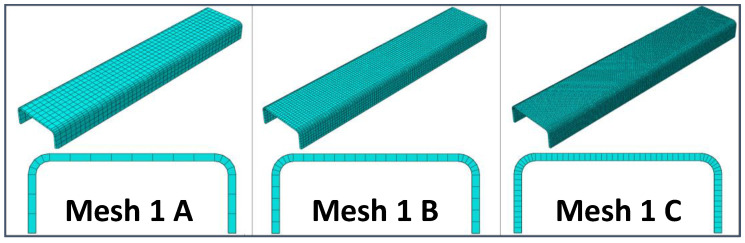
One element in the thickness direction: Coarser mesh (Mesh 1A), intermediate mesh (Mesh 1B), and finer mesh (Mesh 1C).

**Figure 4:**
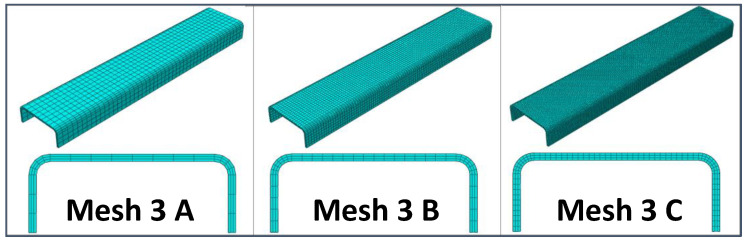
Three elements in the thickness direction: Coarser mesh (Mesh 3A), intermediate mesh (Mesh 3B), and finer mesh (Mesh 3C).

**Figure 5:**
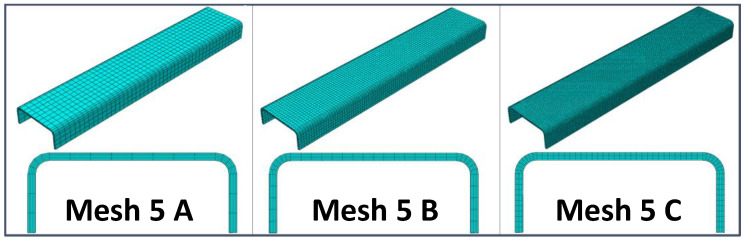
Five elements in the thickness direction: Coarser mesh (Mesh 5A), intermediate mesh (Mesh 5B), and finer mesh (Mesh 5C).

A preliminary mesh convergence analysis has been carried out to determine the best compromise between computational costs and accuracy of the results in terms of predicted stiffness. Hence, different static linear analyses have been performed, varying the in-plane and through-the-thickness element sizes. In particular, three different mesh element sizes were considered: Coarser (8 mm), intermediate (4 mm), and finer (2 mm). Moreover, for each in-plane element size, three through-the-thickness mesh configurations have been investigated, resulting in the nine mesh configurations that have been analyzed and compared to each other. In particular, Figure 3, Figure 4 and Figure 5 report the model configuration considering, respectively, one, three, and five elements through the thickness. Eight-node Abaqus Continuum shell elements SC8R [34], with 3 degrees of freedom (DoF) per node and a reduced integration scheme, have been used to discretize the model.

**Figure 6:**
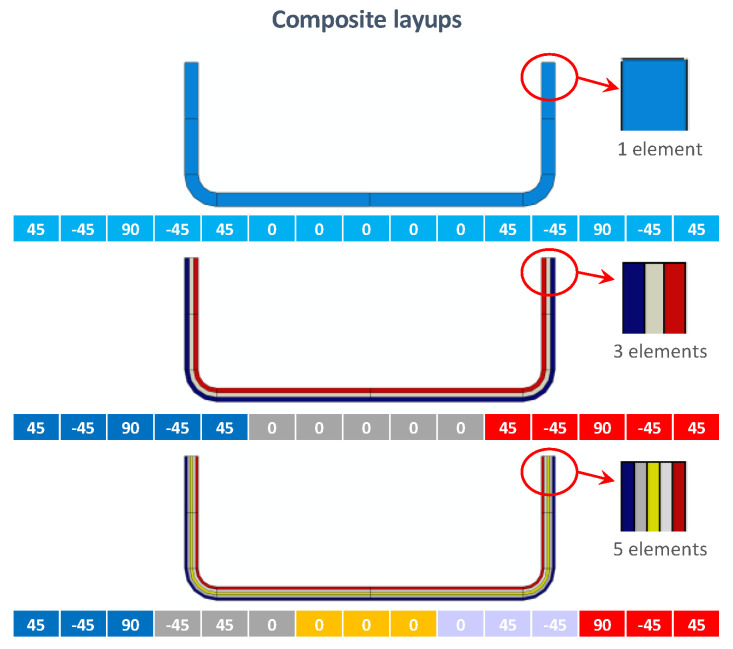
Composite layups.

The different composite layups, which depend on the number of elements in the thickness direction, are shown in Figure 6.

A linear static compression test has been performed on the configurations, to compare the results in terms of stiffness and computational time (16-core Intel Xeon E5-2687W 3.10 GHz processor, 64 GB RAM). The results are summarized in Table 2.

According to the results reported in Table 2, the configuration 3B represents the best compromise between accuracy of the results and computational time. Hence, this model will be used in all the subsequent analyses.

### 3.2. Validation of the Numerical Model

The selected numerical model has been validated by comparison with experimental test campaign results, in terms of stiffness and failure. In particular, two experimental tests have been performed:

- Experimental Test T1: Compressive test aimed to determine the stiffness of the structure;
- Experimental Test T2: Compressive test up to the total failure.

**Figure 7:**
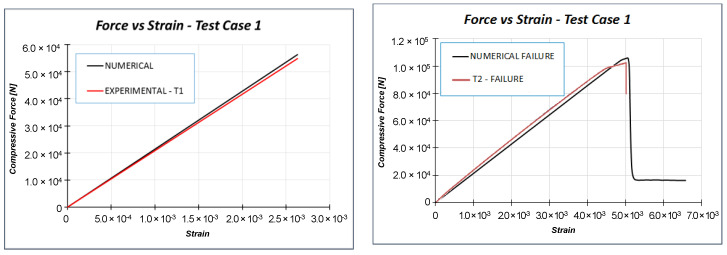
Numerical-experimental comparisons: Load vs. strain.

**Figure 8:**
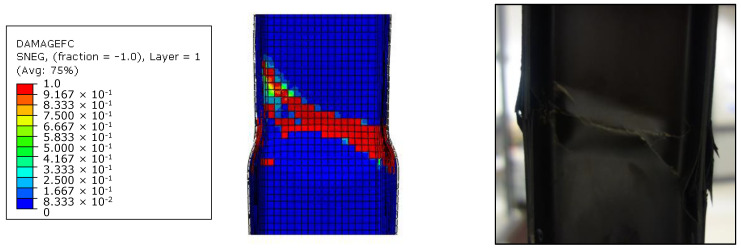
Numerical-experimental comparisons: Failures.

In Figure 7 and Table 3, it is possible to appreciate the good agreement between the numerical predicted solutions and the results of the experimental campaign tests in terms of stiffness and failure load. Moreover, the failure mode of the test article, computed by using the Hashin’s failure criteria, has also been predicted with a high level of accuracy, as highlighted in Figure 8. No buckling phenomena occur up to the failure load both experimentally and numerically.

### 3.3. Dynamic Buckling Analysis

**Figure 9:**
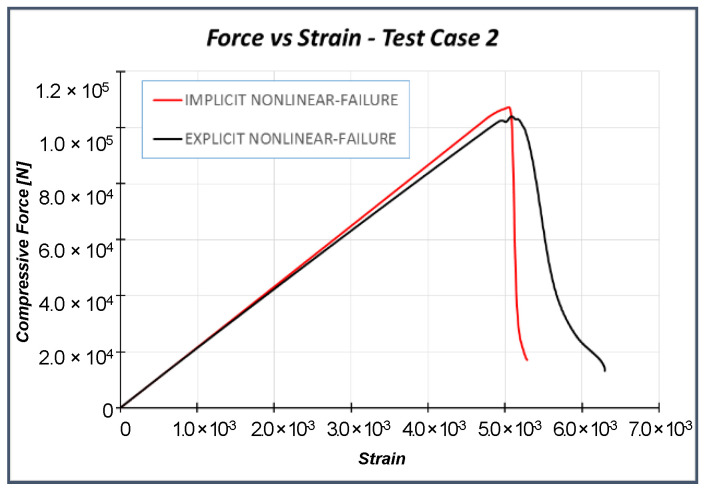
Implicit–explicit formulation comparisons: Load vs. strain.

**Figure 10:**
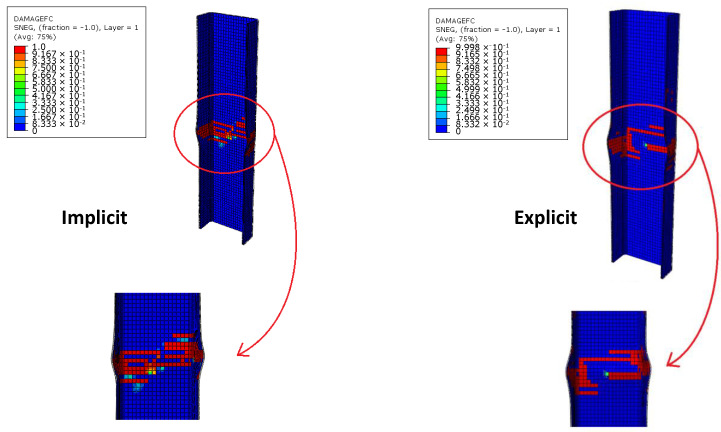
Implicit–explicit formulation comparisons: Failures.

Once the numerical model has been validated, explicit numerical analyses [35,36] have been performed on Test Case 2 to investigate the arising of the dynamic buckling phenomenon. However, as a preliminary step, compressive tests have been performed on Test Case 2 by using implicit and explicit formulations, and the results have been compared in order to assess the robustness of the numerical model and the influence of the solver’s scheme. Experimental data are available only for quasi-static loading conditions and, therefore, for implicit solutions. As the numerical investigations presented in the next sections have been performed with an explicit solver, the implicit-explicit comparison aims to investigate the possible influence of the solution scheme on the results. In the explicit analysis, the load is applied by a smoothed step in order to minimize the inertial effect (related to dynamic simulation). Thus, no significant differences are expected; anyway, in order to highlight this aspect, the implicit–explicit comparison is presented, to demonstrate that differences in structural response, in the explicit model, are related only to different loading conditions and cannot be addressed to the adopted solution scheme. Both models were discretized by means of continuum shell elements (SC8R). All explicit analyses have been performed setting a structural damping equal to 2%. Good agreement has been found between the solutions of both formulations, as confirmed by the load–strain chart in Figure 9, the element failures in Figure 10, and the failure displacements and loads in Table 4.

According to Figure 9 and Figure 10, no relevant differences between the implicit and explicit solution can be found. The slight deviation in the failure path is related to small differences in the load distribution, due to the difference in the calculation of nodal displacements and forces between the two solution schemes. Indeed, the results must not be focused on the number of failed elements (Figure 10) but, instead, on the global behavior, which is reported in the graph of Figure 9. The buckling load of the structure is very close to the failure load, so no post-buckling regime is expected. The slight deviation between the two curves (at about 3.0 × 10^−3^ strain) is related to the damage propagation, not to the post-buckling regime. In the explicit analysis, the elements reach the total failure state faster with respect to the implicit one, leading to an earlier and smaller loss in stiffness.

**Figure 11:**
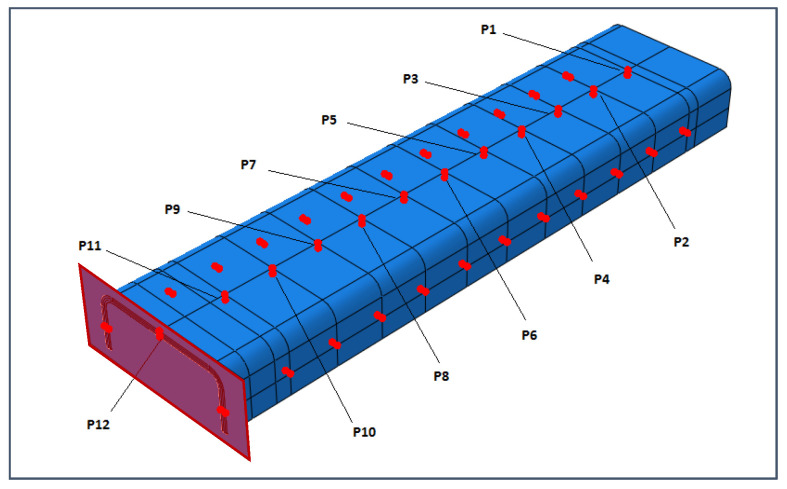
Explicit analysis: Rigid plate and control points.

In the dynamic explicit analyses, a rigid plate has been used to apply the time-dependent load onto the structure as a controlled displacement, as shown in Figure 11, which reports the control points where the outputs have been monitored as well. Seventy-two control points have been used to evaluate the occurrence of the buckling phenomena; they are placed along the entire stanchion and in a different section location in the back-to-back configuration.

Furthermore, several numerical element formulations have been investigated, to assess the capability of different numerical formulations to simulate the dynamic phenomenon. Hence, alongside the aforementioned continuum shell (SC8R) element discretization with three elements in the thickness direction (Model 3B), the following models, summarized in Table 5, have been introduced:

- Model S: This model has been discretized by using four-node linear shell (S4) elements [34]. The same in-plane element size of Model 3B has been considered; however, obviously, no division in the thickness direction has been employed. The equivalent properties of the entire laminate, reported in Table 6, have been used for each element.
- Model 1R: This model has been discretized by using eight-node linear solid elements with a reduced integration scheme (C3D8R) [34]. The same in-plane mesh discretization used for Model 3B and Model S has been considered. One element has been placed in the thickness direction, in order to avoid the use of the layered option. The equivalent mechanical properties of the entire laminate (Table 6) have been assigned to the elements.
- Model 3R: This model is similar to model 1R but differs from the latter as three elements in the thickness direction have been considered. Hence, the properties of the equivalent five-plies sublaminates corresponding to those shown in Figure 6 (Table 7) have been properly assigned to each element.

**Figure 12:**
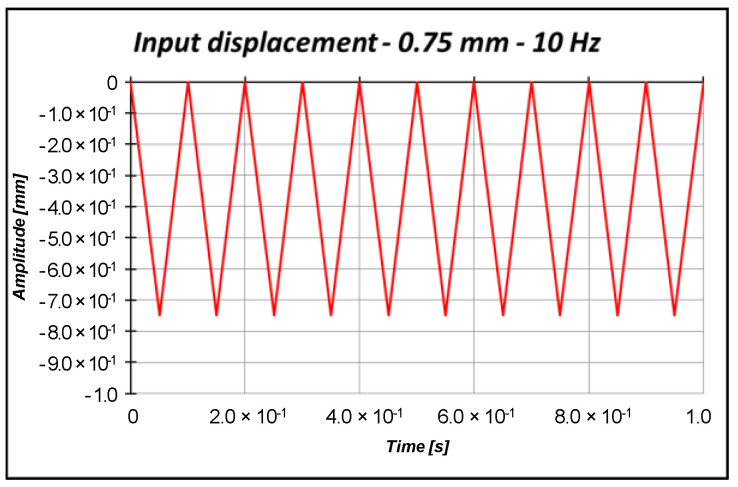
Input triangular displacement.

**Figure 13:**
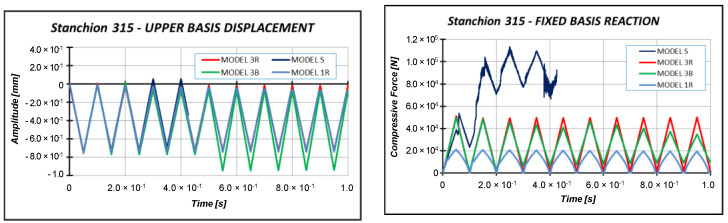
Free side displacement and fixed side reaction comparisons.

The numerical models have been subjected to a triangular load with a maximum compressive amplitude equal to 0.75 mm (smaller than critical displacement estimated by numerical analysis, which is about 1.53 mm), shown in Figure 12. Then, the numerical models have been compared in terms of the free side displacements and fixed side reactions reported in Figure 13.

The results reported in Figure 13 demonstrated that the most suitable numerical model able to simulate a dynamic compressive analysis is model 3R, which has been selected in the subsequent analyses. Indeed, in model 3B, the displacement of the specimen is equal to the compressive displacement imposed on the plate only in the initial stage of the load cycle, up to t = 0.25 s. Then, the displacements start to differ from each other. This phenomenon is even more clear starting from t = 0.5 s. Moreover, the specimen discretized by model 3B is not able to completely be unloaded after 0.2 s, according to the results of the fixed side reaction. Model S experienced convergence issues, as highlighted by the results in terms of fixed side reactions, which are very irregular. Model 1R experiences issues in terms of free side displacement very similar to those encountered by Model 3B. Moreover, Model 1R is strongly affected by hourglass, as highlighted by fixed side reactions.

Sinusoidal cyclic displacements have then been applied on model 3R. According to preliminary eigenvalue buckling analyses, not reported here for the sake of brevity, the critical Test Case 2 displacement is 1.53 mm. Hence, three input displacements, characterized by a sinusoidal shape with a 10 Hz frequency, have been considered:

- Applied displacement = 1.0 mm (below the critical displacement);
- Applied displacement = 1.53 mm (equal to the critical displacement);
- Applied displacement = 2.0 mm (above the critical displacement).

**Figure 14:**
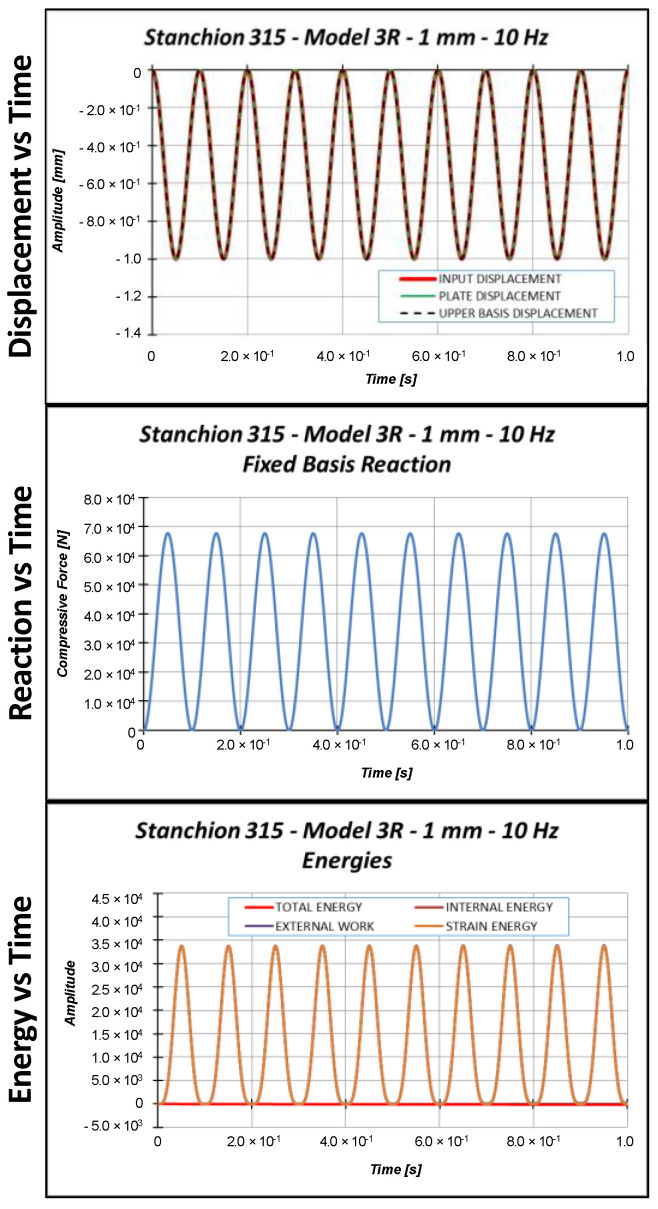
Displacement, reaction, and energies as a function of time: 1.0 mm applied displacement.

**Figure 15:**
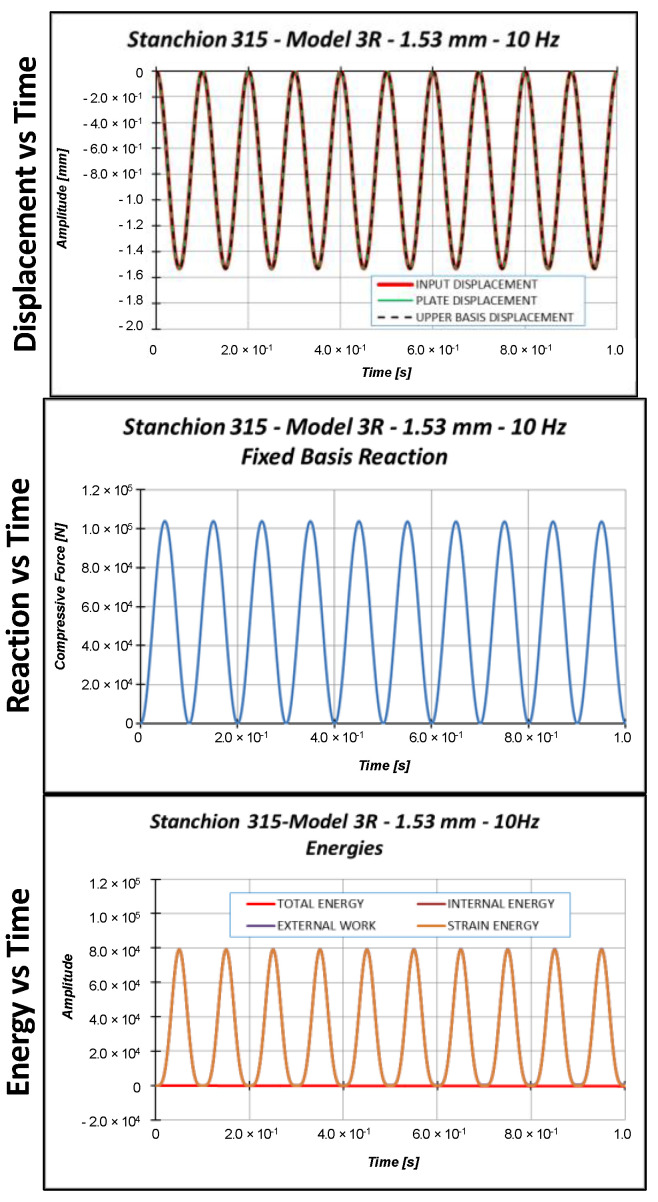
Displacement, reaction, and energies as a function of time: 1.53 mm applied displacement.

**Figure 16:**
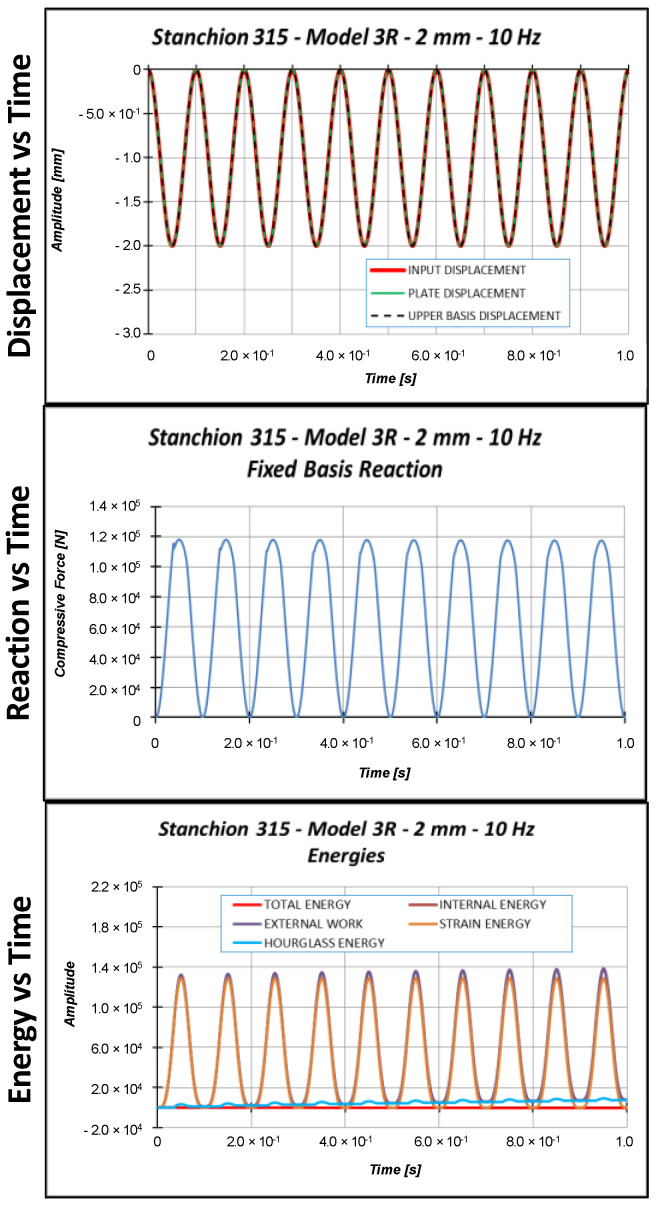
Displacement, reaction, and energies as a function of time: 2.0 mm applied displacement.

Figure 14, Figure 15 and Figure 16 report the displacements, the reactions, and the energies, as a function of time, for each load case.

**Figure 17:**
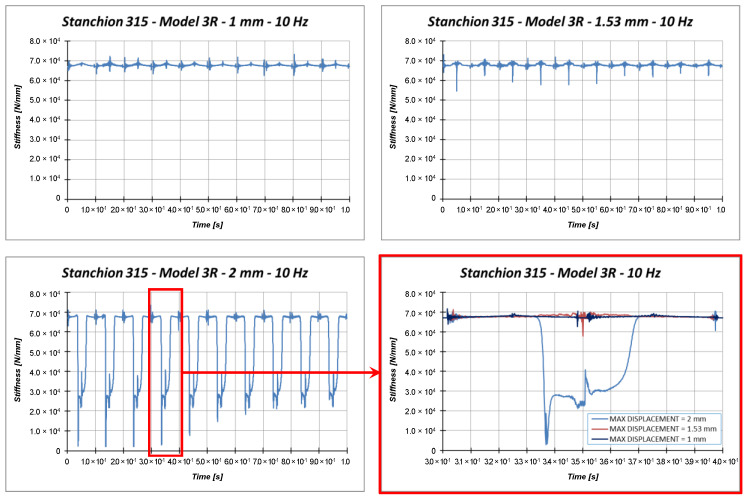
Model 3R: Stiffness comparisons.

More detailed information on the dynamic behavior of the investigated test case can be obtained from Figure 17, which compares the stiffness corresponding to each load amplitude.

According to Figure 17, the stiffness of the model loaded by a maximum displacement equal to 1 mm is almost constant, meaning that no buckling occurs. The stiffness of the specimen loaded with a maximum amplitude equal to 1.53 mm (static buckling load) experiences a clear variation in correspondence to the maximum compression. However, this variation is almost instantaneous; hence, it cannot be considered representative of the buckling phenomenon. These considerations cannot be drawn for the 2 mm applied displacement. Indeed, in this last load case, the stiffness variation is more pronounced and lasts longer in time. Considering the provided results, it is quite clear that the applied load velocity (about 2 × maximum displacement × frequency) is too small to generate any resonance issue, which could lead to instability phenomena. Further, a constant structural damping (0.2%) has been adopted; hence, small slowdown effects on the elastic wave propagation can be observed with respect to the quasi-static case.

**Figure 18:**
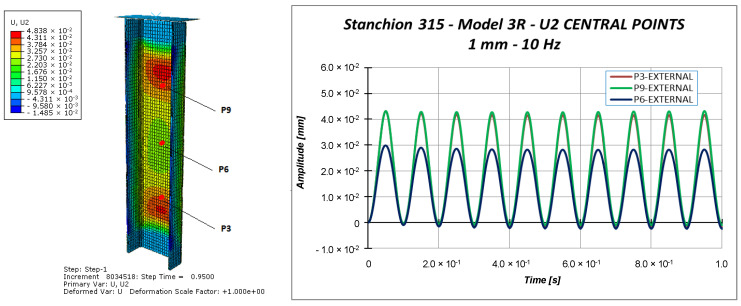
Out-of-plane displacement: 1.0 mm applied displacement.

**Figure 19:**
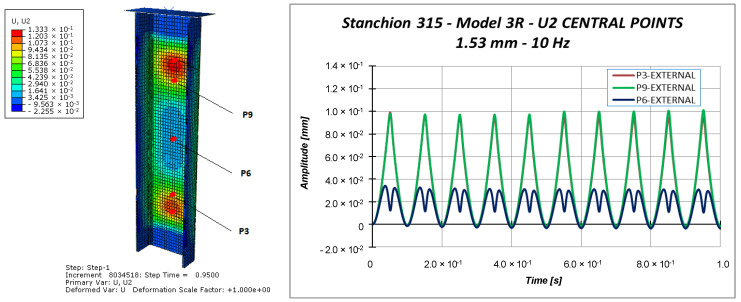
Out-of-plane displacement: 1.53 mm applied displacement.

**Figure 20:**
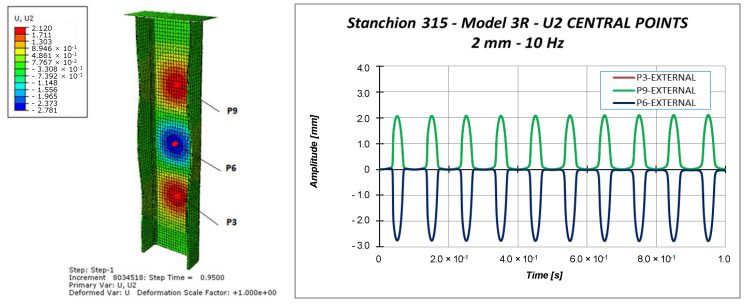
Out-of-plane displacement: 2.0 mm applied displacement.

Other information can be taken considering the out-of-plane displacements U2 of the central points of the specimen (points P3, P6, and P9 in Figure 11), reported in Figure 18, Figure 19 and Figure 20.

Indeed, Figure 18, Figure 19 and Figure 20 highlight the symmetrical out-of-plane displacement patterns resulting from the structural response of these symmetric systems (due to the adopted material formulation), as suggested in [37].

**Figure 21:**
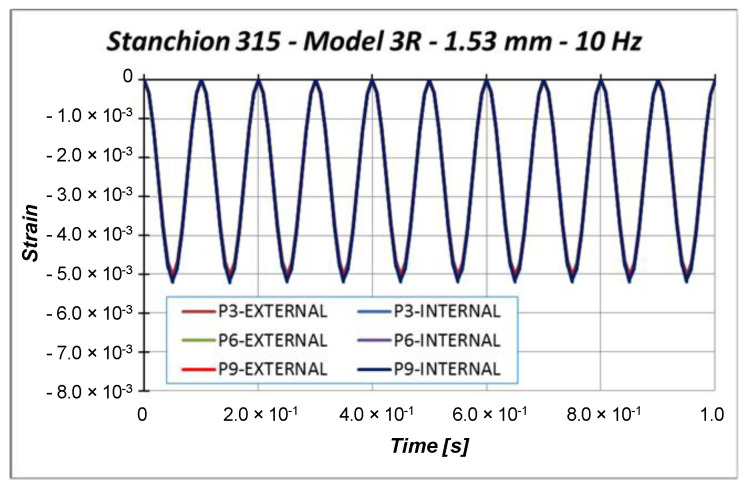
Strain: 1.53 mm applied displacement.

**Figure 22:**
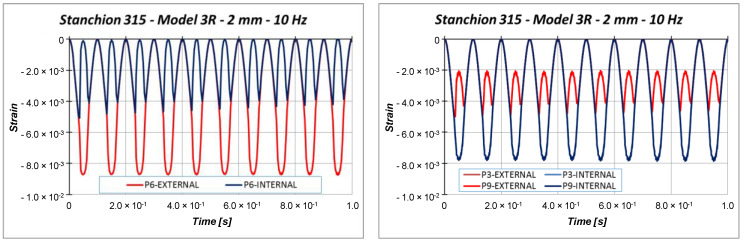
Strain: 2.0 mm applied displacement.

Considering a maximum applied displacement equal to 1 mm (Figure 18), no buckling occurs and all the points experience minimal out-of-plane displacements, which follows a sinusoidal and regular behavior. In the case of 1.53 mm maximum applied displacement (Figure 19), a more irregular sinusoidal behavior can be observed. Moreover, point P6 experiences a double peak, corresponding to the maximum compressive displacement, which can be considered representative of the buckling onset. Figure 20 reports the out-of-plane displacements in the case of 2 mm compression. In this case, point P6 experiences a shift in the value of the displacement, due to the buckling. This can be confirmed by monitoring the strains of the internal and external points considering a maximum applied displacement of 1.53 mm (Figure 21) and 2.0 mm (Figure 22).

Indeed, the strains in Figure 21 are almost coincident, proving that compressive strain is mainly due to the compressive applied displacement and is not representative of the buckling phenomenon. On the other side, Figure 22 shows that the external point P6 experiences a compressive strain variation, while the internal point P6 experiences a tensile strain variation, which clearly indicated that buckling occurs. The strains’ divergence (outer/inner control point), which indicates the buckling onset, occurs at 76.6% of the maximum amplitude, which corresponds to a displacement very close to the critical one. Further, with the increment in cycles, the structural behavior is very repetitive, but it is possible to see a small and slow reduction in the critical displacement.

## 4. Conclusions

In this work, the structural behavior of a composite floor beam subjected to cyclical low-frequency compressive loads has been presented. Sensitivity analyses have been carried out in order to find the most suitable numerical model able to simulate the dynamic analyses, considering different mesh discretization, element, and section formulations. The numerical model has been validated with respect to experimental data obtained under quasi-static loading conditions, thus adopting the implicit formulation. In order to investigate the influence of the solution scheme on the numerical results, an explicit–implicit comparison has been performed. The results highlighted that the adopted technique (smoothed step) allows us to minimize the inertial effects and, thus, to have a good agreement between the two solver schemes. Therefore, the explicit model can also be considered indirectly validated. Further, considering that the applied loading velocity, in dynamic cyclic loading conditions, is very small (lower than 0.1 m/s), the previous consideration can be extended to the final model.

Explicit non-linear analyses have been performed, applying dynamic 10 Hz frequency compressive loads. Load cycles with different amplitudes have been considered: 1 mm (about 70% of the static buckling value), 1.53 mm (close to the static buckling value), and finally 2 mm (above the static buckling value).

The results showed that low-frequency cyclic loads (compared to the test article’s own frequencies, that are in the order of hundreds Hz) with peak values lower than the static buckling load value are not capable of triggering significant instability. This behavior is confirmed by comparing the stiffness of the models as a function of time for the different load levels applied at the same 10 Hz frequency.

In particular, all reported results highlight that the applied load velocity (obtained from the load frequency) is small enough to not generate any resonance issue; therefore, the deformation elastic wave is able to cover the entire specimen length in a time smaller than that related to a single load cycle. Thus, considering the applied load frequency, no significant differences were observed with respect to the static case. All of this will be confirmed by future experimental activities in which the frequency-damping dependency will also be investigated (also beyond the applied frequency). This parameter, assumed constant in this work, could generate a slowdown in the propagation of the elastic wave and, thus, anticipate resonance effects.

## Figures and Tables

**Table 1:** Mechanical properties of the lamina.

ρ [g/cm^3^]	th [mm]	E_11_ [MPa]	E_22_ [MPa]	G_12_ [MPa]	G_13_ [MPa]	G_23_ [MPa]	ν_12_ [-]	X_t_ [MPa]	X_c_ [MPa]	Y_t_ [MPa]	Y_c_ [MPa]	S_c_ [MPa]
1.6	0.186	135,000	8430	4160	4160	3328	0.26	2257	800	75	171	85

**Table 2:** Mesh convergence analysis results.

Number of Elements in the Thickness	Element Size	Model Name	Number of Elements (Total)	Stiffness [kN/mm]	Computational Time
1	8 mm	Mesh 1A	1056	56.220	10 s
	4 mm	Mesh 1B	3572	56.317	15 s
	2 mm	Mesh 1C	12,920	56.340	21 s
3	8 mm	Mesh 3A	3168	56.215	13 s
	4 mm	Mesh 3B	10,716	56.305	18 s
	2 mm	Mesh 3C	38,760	56.327	33 s
5	8 mm	Mesh 5A	5280	56.097	16 s
	4 mm	Mesh 5B	17,860	56.180	21 s
	2 mm	Mesh 5C	64,600	56.192	52 s

**Table 3:** Numerical-experimental comparisons: Stiffness and failure load.

	Stiffness	Failure Load
**Experimental**	54.8 kN/mm	103.7 kN
**Numerical**	56.3 kN/mm	106.0 kN
**Error**	2.6%	2.2%

**Table 4:** Implicit–explicit formulation comparisons: Failure displacement and failure load.

	Failure Displacement	Failure Load
**Implicit**	1.59 mm	107.3 kN
**Explicit**	1.60 mm	104.0 kN
**Error**	0.06%	3.1%

**Table 5:** Investigated element formulations.

Model Name	Element Type	# of Elements in the Thickness	Laminate Type
Model 3B	Continuum Shell (SC8R)	3	Composite Layup
Model S	Shell (S4)	1	Composite Layup
Model 1R	3D Stress (C3D8R)	1	Equivalent Laminate
Model 3R	3D Stress (C3D8R)	3	Equivalent Laminate

**Table 6:** Equivalent laminate mechanical properties.

th [mm]	E_11_ [MPa]	E_22_ [MPa]	E_33_ [MPa]	G_12_ [MPa]	G_13_ [MPa]	G_23_ [MPa]	ν_12_ [-]
2.79	60,267	37,845	8430	20,559	20,559	3328	0.43

**Table 7:** Equivalent sublaminate mechanical properties.

Stacking Sequence	th [mm]	E_11_ [MPa]	E_22_ [MPa]	E_33_ [MPa]	G_12_ [MPa]	G_13_ [MPa]	G_23_ [MPa]	ν_12_ [-]	ν_13_ [-]	ν_23_ [-]
[0]_5_	0.93	135,000	8430	8430	4160	4160	3328	0.26	0.3	0.3
[−45, 45, 90, 45, −45]	0.93	22,674	39,437	8430	28,759	28,759	3328	0.44	0.3	0.3
